# Supplementary material for: ARE/Nrf2 Transcription System Involved in Carotenoid, Polyphenol, and Estradiol Protection from Rotenone-Induced Mitochondrial Oxidative Stress in Dermal Fibroblasts
Source: Antioxidants (Basel). 2024 Aug 21;13(8):1019. doi: 10.3390/antiox13081019 (PMC11351643; doi:10.3390/antiox13081019)
Supplement: Supplementary file 1 [file antioxidants-13-01019-s001.zip › antioxidants-3134174-supplementary.pdf]

Supplementary Materials

Rotenone Triggers Cell Death of Dermal Fibroblasts

The number of dermal fibroblasts was measured using crystal violet staining after treatment with the indicated concentrations of rotenone for 48 h. There were no significant differences in the reduction in cell number by the tested concentrations, but the reproducibility of the results was better at 1 μM.

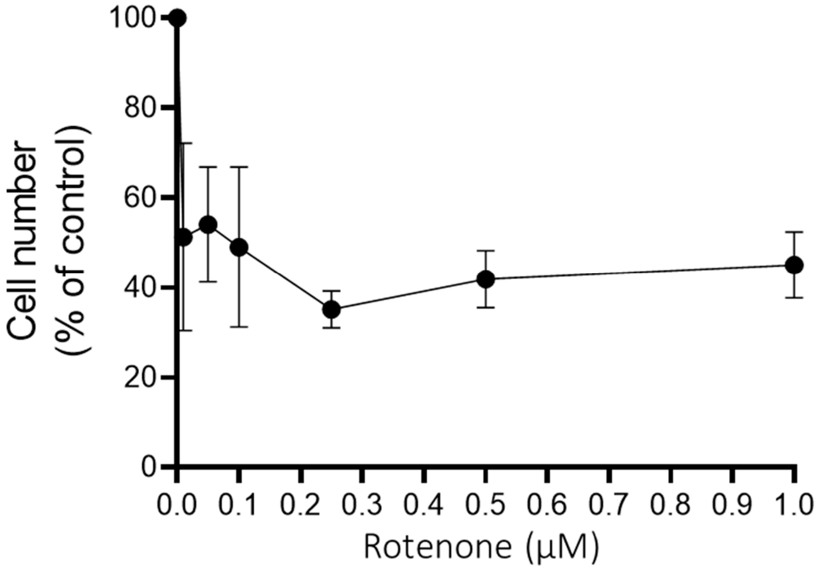

Figure S1. Reduction of cell number by rotenone is not dose-dependent. Normal Human Dermal Fibroblasts (NHDF) were seeded in 96-well plates at a density of 5×10^3 cells/well in DMEM-DCC-FBS medium, and 48 h later, the medium was replaced with one containing the indicated concentrations of rotenone, and incubated for an additional 48 h. Thereafter, the medium was removed, and the cell number was determined using a crystal violet assay (Sigma-Aldrich, Rehovot, Israel), according to the manufacturer's instructions. Results are presented as percent of the control without rotenone and the treatment compounds. One hundred percent of the cell number was 34,730 ± 4253 cells/well. Values are the means ± SEM of three to five experiments, each performed in triplicate.
